# Review of Recent Developments on Using an Off-Lattice Monte Carlo Approach to Predict the Effective Thermal Conductivity of Composite Systems with Complex Structures

**DOI:** 10.3390/nano6080142

**Published:** 2016-07-30

**Authors:** Feng Gong, Hai M. Duong, Dimitrios V. Papavassiliou

**Affiliations:** 1 School of Energy Science and Engineering, University of Electronic Science and Technology of China, Chengdu 610000, China; gongfeng@u.nus.edu; 2 School of Chemical, Biological, and Materials Engineering, University of Oklahoma, Norman, OK 73019, USA; 3 Department of Mechanical Engineering, National University of Singapore, Singapore 117576, Singapore; mpedhm@nus.edu.sg

**Keywords:** off-lattice Monte Carlo simulation, multiphase polymer composites, carbon nanotube, graphene, thermal conductivity

## Abstract

Here, we present a review of recent developments for an off-lattice Monte Carlo approach used to investigate the thermal transport properties of multiphase composites with complex structure. The thermal energy was quantified by a large number of randomly moving thermal walkers. Different modes of heat conduction were modeled in appropriate ways. The diffusive heat conduction in the polymer matrix was modeled with random Brownian motion of thermal walkers within the polymer, and the ballistic heat transfer within the carbon nanotubes (CNTs) was modeled by assigning infinite speed of thermal walkers in the CNTs. Three case studies were conducted to validate the developed approach, including three-phase single-walled CNTs/tungsten disulfide (WS_2_)/(poly(ether ether ketone) (PEEK) composites, single-walled CNT/WS_2_/PEEK composites with the CNTs clustered in bundles, and complex graphene/poly(methyl methacrylate) (PMMA) composites. In all cases, resistance to heat transfer due to nanoscale phenomena was also modeled. By quantitatively studying the influencing factors on the thermal transport properties of the multiphase composites, it was found that the orientation, aggregation and morphology of fillers, as well as the interfacial thermal resistance at filler-matrix interfaces would limit the transfer of heat in the composites. These quantitative findings may be applied in the design and synthesis of multiphase composites with specific thermal transport properties.

## 1. Introduction

Polymer composites can combine the merits of fillers and polymer matrix in a hybrid system. They can even replace the traditional materials (e.g., glass-based materials, metals and alloys) in a number of applications, due to their unique optical, mechanical, thermal and electrical properties [1]. During recent years, carbon-based two-phase polymer composites, such as carbon nanotube (CNT)-polymer composites and graphene-polymer composites, have attracted much attention from both academy and industry, owing to their superior strength, high thermal conductivity and good electrical conductivity [2,3,4]. In general, the thermal conductivity and electrical conductivity of carbon additives (e.g., CNTs, graphene, carbon black, carbon fiber) are both much higher than those of polymer matrices. 

With the additions of carbon fillers into polymers, both the thermal and electrical conductivity of composites can be significantly enhanced, which may limit the applications of composites in some specific fields. For instance, in electronic packaging, the packaging materials require high thermal conductivity but low electrical conductivity. Two-phase composites with only one type of carbon filler are difficult to achieve this requirement. Multi-types of fillers are required to enhance thermal conductivity but retain electrical insulation for composites. For example, Dai et al. developed polyimide (PI) composites containing one-dimensional (1D) SiC nanowires and two-dimensional (2D) graphene nanosheets as hybrid fillers [5]. As the 1D SiC nanowires prevent the graphene nanosheets forming networks, the three-phase composite achieves a low electrical conductivity (~1.32 × 10^−10^ S/cm) but a high thermal conductivity (~0.577 W/m·K) [5].

On the other hand, as electronic devices are developing to the integrated and micro-scale level, thermal interface materials (TIMs) with high thermal conductivity are necessary to effectively dissipate the heat and then to protect electronic devices [6,7]. Traditional TIMs with one type of filler require high loading of fillers to achieve the expected thermal conductivity of 0.5–5 W/m·K, which dramatically increases the cost and the viscosity of the composite. Recently, it has been found that combining multiple fillers in polymer may significantly enhance the thermal conductivity of the composites owing to the synergistic effect of fillers. Zhao et al. obtained a three-phase composite by adding 0.2 vol % graphene foam (GF) and 2.7 vol % multilayer graphene flakes (MGFs) into polydimethylsiloxane (PDMS). Due to the synergistic effect of GF and MGFs, the MGF/GF/PDMS composites achieved a thermal conductivity of up to 1.08 W/m·K, which is 440%, 184% and 80% higher than those of pure PDMS, 0.2 vol % GF/PDMS and 2.7 vol % MGF/PDMS composites, respectively [8]. 

In addition to the above multiphase composites, other diverse types of multiphase composites have been developed for their specific applications, such as CNT/inorganic nanoparticle/polymer [9,10], CNT/graphene/polymer [11,12,13], graphene/inorganic nanoparticle/polymer [14,15], and polymer blends [16,17]. Such multiphase composites can combine the merits of fillers and matrix to achieve advanced properties for diverse applications. Experimental measurements have shown that the effective thermal conduction properties of multiphase composites can be significantly enhanced due to the synergistic effects of fillers. However, computational studies in this field are still limited due to the complex structure and composition of multiphase composites. The effective medium theory has been used to estimate the effective thermal conductivity (*K*_eff_) of multiphase composites [18]. The *K*_eff_ is calculated in separated steps, which neglects the synergistic effect of fillers. An effective modeling of heat transfer in multiphase composites can reveal the thermal transport phenomena and limitations in them, which may help to design multiphase composites with specific thermal properties. 

In this article, we review an effective approach to model the heat transfer in multiphase composites with complex structures based on our work about the computational studies on the effective thermal properties of multiphase composites. The paper is organized as following: In Simulation Methods, an off-lattice Monte Carlo approach for modeling heat transfer in a three-phase composite is presented in detail. Three case studies are discussed in Results and Discussion to demonstrate the novelty, accuracy and superiority of the developed approach. The case-study systems include three-phase single-walled CNT (SWNT)/tungsten disulfide (WS_2_)/poly(ether ether ketone) (PEEK) composite, four-phase SWNT/SWNT bundle/WS_2_/PEEK composite, and complex graphene/poly(methyl methacrylate) (PMMA) composite. We quantitatively studied the factors influencing the thermal transport in multiphase composites, such as the morphology, dispersion, alignment and concentration of fillers, as well as the effects of the interfacial thermal resistance between any pair of components. Such quantitative findings may guide researchers to design and synthesize multiphase composites with specific thermal transport properties. 

## 2. Simulation Methods

**Figure 1:**
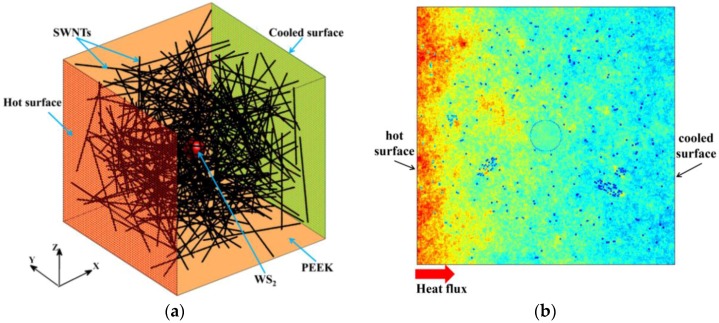
A schematic plot of the SWNT/WS_2_/PEEK model: (**a**) a WS_2_ nanoparticle (110 nm diameter) is placed in the center of a PEEK cube with a side length of 925 nm, while 317 SWNTs (2 nm diameter and 500 nm length) are randomly distributed in the PEEK cube. The WS_2_ particle is painted red and the nanotubes are black in the figure. Constant heat flux is applied along the *x* direction by creating a hot surface and a cooled surface (Reproduced with permission from [18]. Copyright Elsevier, 2015); (**b**) a contour plot of thermal walker distribution in the center *xy* plane of the SWNT/WS_2_/PEEK model at the thermal steady state (Reproduced with permission from [24]. Copyright American Chemical Society, 2015).

In this section, we chose a three-phase SWNT/WS_2_/PEEK composite to illustrate the off-lattice Monte Carlo approach. The off-lattice Monte Carlo approach regards the heat flow as the result of the movement of discrete thermal walkers with random motion [19]. The Monte Carlo approach has been successfully applied to model heat or mass transport in flow through porous materials [20,21] and in convective flows [22,23]. A three-dimensional (3D) model was built based on the SWNT/WS_2_/PEEK composite fabricated by Naffakh et al. [9]. As shown in Figure 1a, SWNTs (500 nm length and 2 nm diameter) and WS_2_ spherical nanoparticles (110 nm diameter) were randomly and uniformly dispersed in PEEK matrix (925 nm side length). The mass fractions of SWNTs, WS_2_ nanoparticles and PEEK were 0.5 wt. %, 0.5 wt. % and 99.0 wt. %, respectively. The 3D model is a representative volume element (RVE) of the three-phase composite, which can be repeated to accurately replicate the realistic composite samples. 

To model the heat transfer in the composite, a constant heat flux was applied along x-direction by continuously releasing a large quantity (e.g., 40,000) of thermal walkers from both sides in each time step (i.e., hot walkers from *x* = 0, and cold walkers from the other side). The two sides can be treated as a hot and a cold surface, as presented in Figure 1a. The hot walkers and cold walkers (with negative energy) have same absolute value of energy, so the energy of the whole system is conserved. A thermal walker jumps randomly following a Brownian motion after released from the surfaces. The Brownian motion is described by position changes of thermal walkers in each direction. The position changes take values from a normal distribution with a zero mean and a standard deviation, σ, expressed as [25]:

(1) $$\sigma =\sqrt{D_{m}\Delta t}$$

where $D_{m}$ is the thermal diffusivity of the matrix and Δt is the time step duration of the simulation. Thermal walkers travel randomly from the initial release surface. Once a thermal walker jumps to the interface between PEEK and a SWNT, it is allowed to either jump into the SWNT with a probability of *f*_m-SWNT_, or still remains in the PEEK matrix with a probability of (1 − *f*_m-SWNT_). The *f*_m-SWNT_ is related to the interfacial thermal resistance (known as *Kapitza* resistance) between the PEEK matrix and SWNTs, which can be estimated from the acoustic mismatch theory, as follows [26]:

(2) $$f_{\text{m-SWNT}}=\frac{4}{\rho_{m}C_{P_{m}}v_{m}R_{\text{bd}}}$$

where $\rho_{m},\;C_{P_{m}},\;v_{m}\;\text{and}\;R_{\text{bd}}$ are the density of the PEEK matrix, the specific heat capacity of PEEK, the speed of sound in PEEK, and the interfacial thermal resistance between PEEK and SWNT, respectively. When a thermal walker jumps into a SWNT, it is assumed to travel with an infinite speed, due to the ballistic phonon transport and ultrahigh thermal conductivity of SWNTs [27]. The implementation of this assumption occurs by randomly placing the walker anywhere within the SWNT. The random placement is based on a uniform distribution function and it occurs in a single time step. The diffusivity inside the CNTs would need to be considered explicitly (using a second Gaussian random motion) if the CNTs were on the same order of magnitude or longer than the wavelength of a phonon. However, this case would present itself for much longer CNTs than the ones consider presently. When inside a SWNT, thermal walkers may exit the SWNT based on another probability, designated as *f*_SWNT-m_. This probability is determined from *f*_m-SWNT_, as [19]:

(3) $$V_{\text{SWNT}}f_{\text{SWNT-m}}=C_{f-\text{SWNT}}\sigma A_{\text{SWNT}}f_{\text{m-SWNT}}$$

where $V_{\text{SWNT}}$ and $A_{\text{SWNT}}$ are the volume and surface area of SWNTs, and $C_{f-\text{SWNT}}$ is a thermal equilibrium factor at the PEEK-SWNT interface which depends on the geometry of SWNTs and the interfacial area between PEEK and a SWNT. The thermal equilibrium factor is introduced in order to preserve the second law of thermodynamics at thermal equilibrium. The above relation between *f*_SWNT-m_ and *f*_m-SWNT_ can be explained as follow: When reaching the thermal steady state, the heat flux exiting a SWNT should be equal to that entering the SWNT. In a time step, all thermal walkers inside a SWNT may travel to the PEEK matrix owing to their infinite speed. However, among the thermal walkers in PEEK, only those around the SWNT surface may jump into the SWNT due to the Brownian motion. In order to maintain a balanced heat flux exiting and entering a SWNT, the relation as described in Equation (3) should be satisfied. 

When inside a WS_2_ nanoparticle, a thermal walker jumps randomly, similar to the way it travels in the PEEK matrix, but with a different thermal diffusivity (that of WS_2_) used in Equation (1). At the PEEK-WS_2_ nanoparticle interface, thermal walkers from either the PEEK or the WS_2_ behave similarly to the walkers crossing the PEEK-SWNT interface from the PEEK side. However, the two probabilities ($f_{{\text{m-WS}}_{2}}$ and $f_{{\text{WS}}_{2}-m}$ ) have different relation from that described in Equation (3), due to the different motion of walkers in WS_2_ nanoparticles and SWNTs. As thermal walkers have Brownian motion in WS_2_ nanoparticles, $f_{{\text{m-WS}}_{2}}$ and $f_{{\text{WS}}_{2}-m}$ are related as:

(4) $$f_{{\text{WS}}_{2}-m}=C_{f-{\text{WS}}_{2}}\frac{{\left(r+\sigma_{m}\right)}^{3}-r^{3}}{r^{3}-{\left(-\sigma_{{\text{WS}}_{2}}\right)}^{3}}f_{{\text{m-WS}}_{2}}$$

where $f_{{\text{WS}}_{2}-m}$ and $f_{{\text{m-WS}}_{2}}$ are the walker travelling probabilities from the WS_2_ nanoparticles to the PEEK matrix and the reverse. Variables *r*, $\sigma_{m}$ and $\sigma_{{\text{WS}}_{2}}$ are the radius of the WS_2_ nanoparticles, the standard deviation of Brownian motion in the PEEK matrix, and the standard deviation of Brownian motion in the WS_2_ nanoparticles, respectively. The parameter $C_{f-{\text{WS}}_{2}}$ is the thermal equilibrium factor at the PEEK-WS_2_ nanoparticle interface, which can be numerically determined in the same manner as $C_{f-\text{SWNT}}$ [27,28,29]. Thermal walkers will be bounced back when they jump outside of the model to maintain a constant heat flux. Periodic boundary conditions are applied in the y and z directions. 

The computational domain was divided into 300 × 300 × 300 grids to calculate the temperature profile, which can be obtained by counting the number of hot walkers and then subtracting the number of cold walkers in a grid cell. Figure 1b shows a typical contour plot of thermal walker distribution in the thermal steady state. With constant heat flux applied along the *x* direction, the temperature along the *x* direction should be a straight line which has a slope inversely proportional to the thermal conductivity of the SWNT/WS_2_/PEEK composite [30]. A reference model of pure PEEK matrix was also built to estimate the effective thermal conductivity (*K*_eff_) of the composite. With the same heat flux and boundary conditions, the temperature profiles along x direction in the composite model and the pure matrix model are related as:

(5) $$q^{\prime \prime }=-K_{\text{eff}}\frac{dT_{c}}{dx}=-K_{m}\frac{dT_{m}}{dx}$$

where *q*″, *T*_c_ and *T*_m_ are the applied constant heat flux, the temperature in the composite and the temperature in the pure PEEK matrix, respectively. *K*_m_ is the thermal conductivity of the pure PEEK matrix, which is known to be 0.23 W/m·K from the literature [31]. Thus, as expressed in Equation (5), the effective thermal conductivity of the composite (*K*_eff_) can be calculated based on the temperature profiles in the composite and the pure PEEK. 

## 3. Results and Discussion

In this section, three case studies are presented and discussed to demonstrate the capabilities of the developed off-lattice Monte Carlo approach. The case study systems include three-phase SWNT/WS_2_/PEEK composites, SWNT/WS_2_/PEEK composites with SWNT bundles and graphene/PMMA composites with different sized graphene sheets. The typical parameters influencing or limiting the thermal transport properties of the composites were quantitatively investigated. The quantitative findings may provide an overview of the thermal transport phenomena and limitation mechanisms in multiphase composites. 

### 3.1. Model of Three-Phase SWNT/WS_2_/PEEK Composites

#### 3.1.1. Validation of the Developed Off-Lattice Monte Carlo Approach

**Figure 2:**
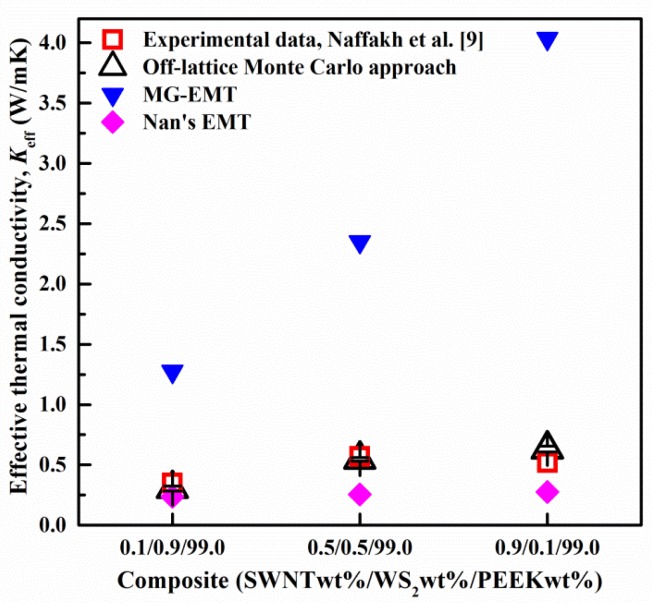
Validation of the developed approach by comparing the simulation results with the experimental data from Reference [9]. The side lengths of the PEEK cubes in 0.1/0.9/99.0, 0.5/0.5/99.0 and 0.9/0.1/99.0 compositions were 760, 925 and 1580 nm, respectively. The interfacial thermal resistance at SWNT-PEEK interface was used as 1.0 × 10^−8^ m^2^·K/W for Nan et al.’s effective medium theory (EMT) [32]. The error bars represent the standard deviation of the results obtained from 3 separate simulations with different distribution of SWNTs. Reproduced with permission from [18]. Copyright Elsevier, 2014.

The developed approach was validated by comparing the simulation results with the measured *K*_eff_ of SWNT/WS_2_/PEEK composites with different compositions [9], as presented in Figure 2. The different compositions (e.g., 0.5/0.5/99.0) were marked with the mass fractions of the SWNTs (0.5%), the WS_2_ nanoparticles (0.5%) and the PEEK matrix (99.0%). In the models for different compositions, the dimensions of the SWNTs and the diameter of the WS_2_ nanoparticle were maintained, while the side length of the PEEK cube was varied. As shown in Figure 2, the simulation results from our approach are in good agreement with the experimental data, which validates the developed approach. 

Effective medium theory approaches (EMT) are commonly applied to predict the *K*_eff_ of composites [33,34,35,36]. For comparison with our developed approach, two widely-used EMTs (i.e., the Maxwell-Garnett model (MG) [35,37] and Nan et al.’s model [38]) were utilized to predict the *K*_eff_ of the SWNT/WS_2_/PEEK composites. The comparison among different models is presented in Figure 2. The MG-EMT overestimated the *K*_eff_ of the SWNT/WS_2_/PEEK composites, which is likely due to the neglect of the interfacial thermal resistance at the SWNT-PEEK interface. On the contrary, Nan et al.’s EMT model underestimated the *K*_eff_ of the SWNT/WS_2_/PEEK composites. This is because that model does not account for the synergistic effects of SWNTs and WS_2_ nanoparticles in the composites. 

#### 3.1.2. Effects of Interfacial Thermal Resistances on the *K*_eff_ of SWNT/WS_2_/PEEK Composites

**Figure 3:**
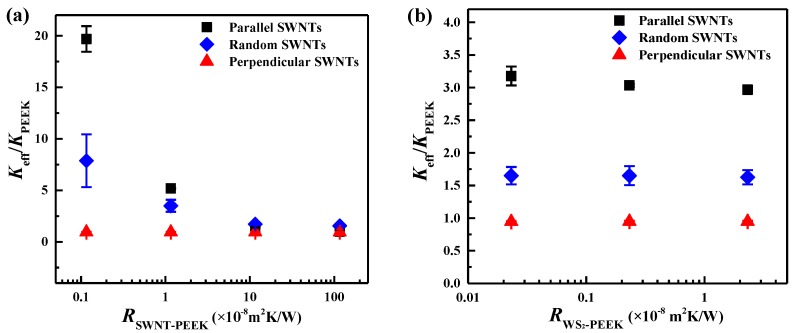
Effects of the interfacial thermal resistances at (**a**) SWNT-PEEK and (**b**) WS_2_-PEEK interfaces on the *K*_eff_ of SWNT/WS_2_/PEEK composites. The 0.5/0.5/99.0 composition was used for this quantitative study. The models with different SWNT orientation (e.g., SWNTs parallel to the heat flux, SWNTs randomly orientated to the heat flux, and SWNTs perpendicular to the heat flux) were built to study the effect of SWNT orientations. The error bars represent the standard deviation of the results from 3 separate simulations with different distribution of SWNTs. Reproduced with permission from [18]. Copyright Elsevier, 2014.

The interfacial thermal resistance (*R*_bd_) at the nanofiller-matrix interface (i.e., SWNT-PEEK, WS_2_-PEEK) is caused by the difference in the vibrational phonon spectra of each side, and by interfacial defects [39,40]. The *R*_bd_ is found to greatly limit the *K*_eff_ of SWNT/WS_2_/PEEK composites [10]. The *R*_bd_ at the SWNT-PEEK ($R_{\text{SWNT-PEEK}}$) and WS_2_-PEEK ($R_{{\text{WS}}_{2}-\text{PEEK}}$) interfaces was varied to investigate the effects on the *K*_eff_ of SWNT/WS_2_/PEEK composites, and the results are presented in Figure 3. The *R*_bd_ is interpreted by an average phonon transmission probability at the interfaces in our approach, as described already above. It has been reported in the literature that the *R*_bd_ in the nanoscale falls into the range of 1.0 × 10^−9^–1.0 × 10^−6^ m^2^·K/W [41,42,43,44], so the $R_{\text{SWNT-PEEK}}$ was varied between 1.158 × 10^−9^ and 1.158 × 10^−6^ m^2^·K/W, which corresponds to an average phonon transmission probability of 0.001–1.0. Similarly, the $R_{{\text{WS}}_{2}-\text{PEEK}}$ ranged from 2.32 × 10^−10^ to 2.32 × 10^−8^ m^2^·K/W, corresponding to an average phonon transmission probability from 0.005 to 0.5.

As shown in Figure 3, the *K*_eff_ of SWNT/WS_2_/PEEK composites decreases with both $R_{\text{SWNT-PEEK}}$ and $R_{{\text{WS}}_{2}-\text{PEEK}}$. In Figure 3b, compared with that in the parallel SWNT case, the effect of $R_{{\text{WS}}_{2}-\text{PEEK}}$ on the *K*_eff_ of SWNT/WS_2_/PEEK composites is much weaker in the random and perpendicular SWNT cases. $R_{\text{SWNT-PEEK}}$ significantly impedes the transfer of heat between SWNTs and the PEEK matrix, weakening the enhancement of SWNTs on the *K*_eff_. At a high $R_{\text{SWNT-PEEK}}$, SWNTs with three different orientations lead to a similar *K*_eff_, which is close to the *K*_PEEK_, indicating that SWNTs do not enhance the heat conduction in the composite. A low $R_{\text{SWNT-PEEK}}$ is desired to obtain the composite with high *K*_eff_, which may be achieved by proper functionalization of SWNTs to couple the phonon spectra of SWNTs and PEEK [45,46,47]. The influence of $R_{{\text{WS}}_{2}-\text{PEEK}}$ on the *K*_eff_ of composites is much weaker than that of $R_{\text{SWNT-PEEK}}$. This may be because: (i) the ultrahigh thermal conductivity of SWNTs allows them to dominate the heat transfer through composites; (ii) the much larger interfacial area of SWNTs make the influence of the SWNT-PEEK interface more significant than that of the WS_2_-PEEK interface for heat transfer; (iii) the long cylindrical SWNTs are more effective than the spherical WS_2_ nanoparticles to enhance the *K*_eff_ of the composites [48], offering a larger characteristic length scale over which heat can be transferred.

#### 3.1.3. Effects of the Morphology of SWNTs on the *K*_eff_ of SWNT/WS_2_/PEEK Composites

**Figure 4:**
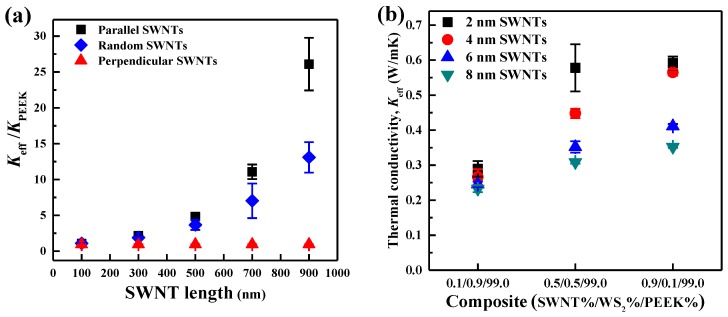
Effects of (**a**) length and (**b**) diameter of SWNTs on the *K*_eff_ of SWNT/WS_2_/PEEK composites. The length was varied from 100 to 900 nm, corresponding to an aspect ratio from 50 to 450. The diameter was varied from 2 to 8 nm while the length was kept as 500 nm. Different compositions with randomly orientated SWNTs were chosen to study the effect of SWNT diameter. The error bars represent the standard deviation of the results obtained from 3 separate simulations with different distribution of SWNTs. Reproduced with permission from [18]. Copyright Elsevier, 2014.

Since SWNTs dominate the heat transfer in the three-phase composites, the morphology of SWNTs (i.e., length and diameter) was varied to study its effect on the *K*_eff_ of SWNT/WS_2_/PEEK composites. The length was varied from 100 to 900 nm while keeping a constant diameter of 2 nm. The diameter was varied from 2 to 8 nm while the length was kept at 500 nm. As presented in Figure 4, when SWNTs are parallel or random to the heat flux, longer SWNTs induce higher *K*_eff_, which is consistent with previous studies [39,49]. Due to the ballistic phonon transport in SWNTs, longer SWNTs are more effective than short ones to transport heat through the composites, leading to a higher *K*_eff_ [50]. For the study of SWNT diameter, a composite with randomly oriented SWNTs was chosen for investigation as it represents most realistic composites. As shown in Figure 4b, the *K*_eff_ of SWNT/WS_2_/PEEK composites increases with the decrease of SWNT diameter, which may be ascribed to the interfacial area of SWNTs. With same volume fraction of SWNTs, a larger interfacial area between SWNTs and PEEK is obtained for SWNTs having smaller diameter. The larger interfacial area can afford more effective heat transfer channels between SWNTs and PEEK, thus inducing a larger enhancement of the *K*_eff_. The diameter used here covers the range of SWNTs (≤2 nm), double-walled CNTs (2–4 nm), and multi-walled CNTs (≥4 nm). Therefore, it can be speculated that SWNTs are more efficient than other CNTs to enhance the *K*_eff_ of SWNT/WS_2_/PEEK composites.

### 3.2. Model of SWNT/WS_2_/PEEK Composites with SWNT Bundles

**Figure 5:**
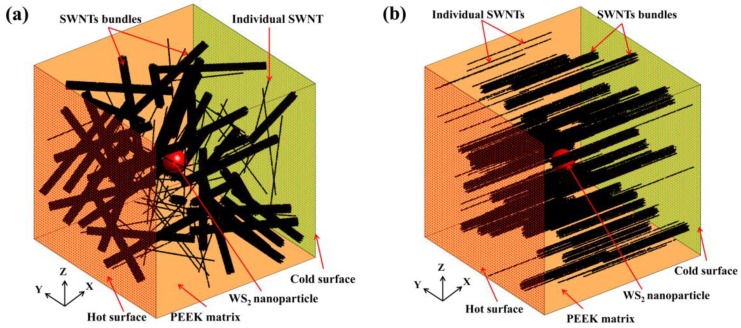
Schematic plot of the SWNT/WS_2_/PEEK model with SWNT bundles: (**a**) A WS_2_ nanoparticle (painted red) with 110 nm diameter is located in the center of a PEEK cube (925 × 925 × 925 nm^3^). A total of 960 SWNTs (2 nm diameter and 500 nm length, painted black) are randomly dispersed in the model, forming 45 bundles with 20 SWNTs in each bundle and 60 unbundled SWNTs. Constant heat flux is applied along × direction; (**b**) composite with individual SWNTs and SWNT bundles oriented parallel to the heat-flux direction. Reproduced with permission from [24]. Copyright American Chemical Society, 2015.

SWNTs tend to aggregate into bundles during the composite synthesis process due to strong van der Waals forces. The thermal conductivity of SWNT bundles is generally lower than that of an individual SWNT in the bundle, due to the interfacial thermal resistance among adjacent SWNTs [51,52,53]. Thus, the SWNT bundles may limit heat transfer in the composites [39]. A SWNT/WS_2_/PEEK composite model with SWNT bundles was built to shed some light on the influence of SWNT bundles on the thermal transport properties of the composites. As shown in Figure 5, there were individual SWNTs, SWNT bundles, WS_2_ nanoparticles, and PEEK matrix in the system. In the present work, only the straight bundles with line contacts were taken into account. The CNT bundles with complicated interconnected networks were out of the scope of this article.

#### 3.2.1. Effects of the Morphology of SWNT Bundles on the *K*_eff_ of SWNT/WS_2_/PEEK Composites

**Figure 6:**
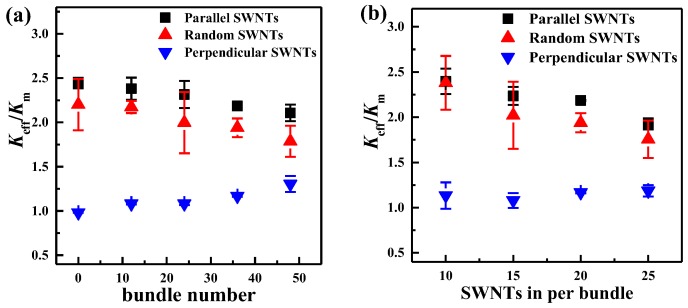
Effects of the morphology of SWNT bundles on the *K*_eff_ of the SWNT/WS_2_/PEEK composites: (**a**) bundle number; and (**b**) the number of individual SWNTs in per bundle. The results for SWNTs with different orientations (parallel, random and perpendicular to the heat flux direction) are all presented. The error bars represent the standard deviation of the results obtained from 3 separate simulations with different distribution of SWNTs and SWNT bundles. Reproduced with permission from [24]. Copyright American Chemical Society, 2015.

The number of SWNT bundles was changed from 0 to 48 (no individual SWNTs) to investigate the effect of SWNT dispersion state on the *K*_eff_ of the SWNT/WS_2_/PEEK composites, and the results are presented in Figure 6a. The models with different SWNT orientations (parallel, random and perpendicular to the heat flux) were all built, as illustrated in Figure 5. As shown in Figure 6a, when SWNT bundles are parallel or randomly oriented relative to the direction of the heat flux, the *K*_eff_ of the composites slightly decreases with an increase of SWNT bundles. This may be caused by the non-uniform distribution of SWNTs with the presence of SWNT bundles, as well as the SWNT-SWNT thermal resistance within the bundles, which prevents heat from conducting along the heat flux direction [54]. Different from the above two SWNT orientations, more SWNT bundles perpendicular to the heat flux can induce a higher *K*_eff_ of the SWNT/WS_2_/PEEK composites. The *K*_eff_ slightly increases by 40% when the bundle number increases to 48. This is likely due to the bigger diameter of SWNT bundles compared with individual SWNTs, which accelerates the heat transfer in the radial direction (also the heat flux direction).

The number of individual SWNTs in each bundle was varied from 10 to 25, at an increment of 5, to study its influence on the *K*_eff_ of the composites. The total number of SWNTs (960) and the number of SWNT bundles (36) were kept constant. As shown in Figure 5b, when SWNT bundles are parallel or randomly oriented relative to the heat flux, more SWNTs in each bundle induce a lower *K*_eff_. With the same number of bundles, more SWNTs per bundle lead to a worse distribution of SWNTs, which may reduce the heat transfer along the heat flux. On the other hand, more SWNTs in a bundle increase the stiffness of the bundle, which weakens the phonon coupling between SWNTs and PEEK via low-frequency vibrations [55], thus leading to a larger SWNT-PEEK thermal resistance [56]. When SWNT bundles are perpendicular to the heat flux, the number of SWNTs in each bundle has no apparent effect on the *K*_eff_ of the composites.

#### 3.2.2. Effects of the SWNT-SWNT Thermal Resistance on the *K*_eff_ of SWNT/WS_2_/PEEK Composites

**Figure 7:**
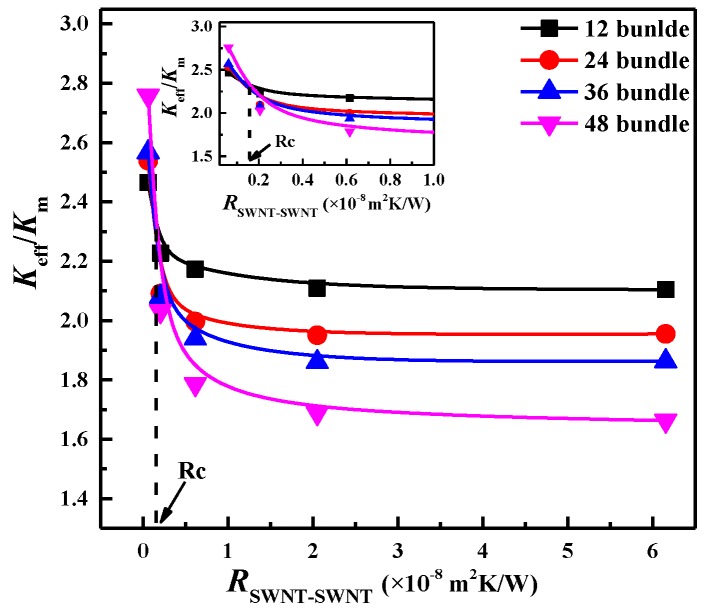
Effect of the SWNT-SWNT thermal resistance on the *K*_eff_ of the SWNT/WS_2_/PEEK composites with 12–48 SWNT bundles. The SWNTs were randomly distributed in the composites. The individual SWNT number in each bundle was kept at 20. The critical TBR (dashed line) was estimated to be *R*_c_ = 0.155 × 10^−8^ m^2^·K/W by intersecting the *K*_eff_ curves of different SWNT bundles, as shown in the insert figure. Reproduced with permission from [24]. Copyright American Chemical Society, 2015.

When SWNTs aggregate into bundle structures, SWNT-SWNT thermal resistance ($R_{\text{SWNT-SWNT}}$) will exist among SWNTs within the bundles. Previous models cannot take into account this resistance for *K*_eff_ prediction, whereas the developed approach can investigate this resistance by controlling the motion of thermal walkers at the SWNT-SWNT interface [24]. The $R_{\text{SWNT-SWNT}}$ was varied from 6.153 × 10^−10^ to 6.153 × 10^−^^7^ m^2^·K/W, corresponding to an average phonon transmission probability of 0.001–1.0 [57]. As presented in Figure 7, the *K*_eff_ of the SWNT/WS_2_/PEEK composites decreases with the increase of the $R_{\text{SWNT-SWNT}}$. A higher $R_{\text{SWNT-SWNT}}$ more greatly prevents heat from transferring among the bundled SWNTs, leading to a lower *K*_eff_ of the composites. 

A critical SWNT-SWNT thermal resistance, $R_{c}$, was found to dominate the heat transfer in the composites, which was estimated to be 0.155 × 10^−8^ m^2^·K/W. When $R_{\text{SWNT-SWNT}}$ < $R_{c}$, more SWNT bundles may induce a higher *K*_eff_ of the composite. This is because at low $R_{\text{SWNT-SWNT}}$, heat prefers to transfer through SWNT-SWNT contacts, thus the SWNT bundles would be more effective heat transfer channels than the mono-dispersed SWNTs. It can be inferred that the detrimental effect of the SWNT bundles could be reduced by decreasing the $R_{\text{SWNT-SWNT}}$ to be less than $R_{c}$, which may be achieved by covalent functionalization to enhance the phonon coupling and weaken the phonon scattering at a SWNT-SWNT interface [58,59]. 

### 3.3. Model of Graphene/PMMA with Complex Structure

In the graphene-based polymer composites, graphene sheets have a large size distribution in the x-y plane (50–500 nm) due to the oxidation and reduction of graphene sheets during the composite fabrication [3]. The existing models commonly ignore the size distribution of graphene sheets, resulting in an inaccurate prediction of the thermal conductivity. In the developed approach, one can take into account the size distribution (various length, width and thickness), volume fraction and orientation of graphene sheets, as well as the interfacial thermal resistance at the graphene-polymer interface. 

In this subsection, a graphene/PMMA composite model was built to validate that the develop approach can be applied to study graphene/polymer composites. Graphene sheets with various length (50–500 nm), width (50–500 nm), thickness (2.4–9.0 nm) and different orientations (parallel, random and perpendicular to the heat flux) were generated in the model. 

**Figure 8:**
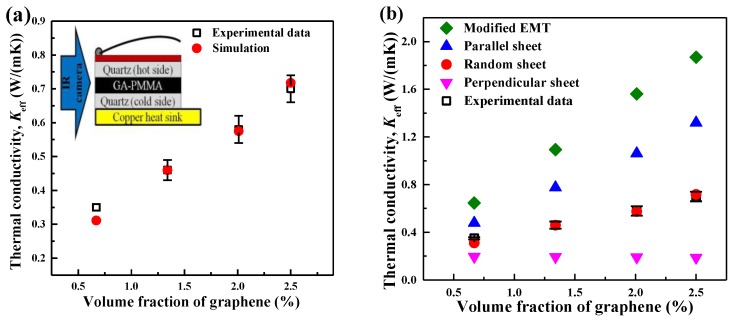
(**a**) Validation of the developed graphene/PMMA model by comparing the simulation results with the experimental data for different volume fractions of graphene sheets (0.67%, 1.34%, 2.01% and 2.50%). The insert figure is the set-up scheme of the comparative infrared microscopy technique for measuring the thermal conductivity. More details of the experimental set-up can be found in Reference [4]; (**b**) the thermal conductivity of GA-PMMA composites as a function of graphene volume fraction. The interfacial thermal resistance between graphene sheets and PMMA was estimated to be *R*_bd_ = 1.906 × 10^−8^ m^2^·K/W in the developed model. The same value was utilized in the composites with parallel- and perpendicular-oriented graphene. In the modified EMT, the utilized thermal conductivity of graphene and the *R*_bd_ of graphene-PMMA were 100 W/m·K and 1.0 × 10^−8^ m^2^·K/W, respectively. The error bars represent the standard deviation of the results from separate measurements of thermal conductivity of graphene/PMMA composites. Reproduced with permission from [4]. Copyright Elsevier, 2015.

The developed graphene/PMMA model was validated by comparing the predicted *K*_eff_ with the measured results, as shown in Figure 8a. As the interfacial thermal resistance (*R*_bd_) at the graphene-PMMA interface was an input value, the model was validated as follows: Estimate the *R*_bd_ by matching the simulated *K*_eff_ with the measured value for one composite, and then use this estimated *R*_bd_ as input to calculate the *K*_eff_ of other composites. The predicted *K*_eff_ showed a good agreement with the experimental data, validating the developed model. The *R*_bd_ was estimated to be 1.906 × 10^−8^ m^2^·K/W. For comparison, a modified effective medium theory (EMT) was used to calculate the *K*_eff_ of the graphene/PMMA composites, and the results are presented in Figure 8b. The estimated *K*_eff_ from the modified EMT is much higher than the experimental results, and even higher than the predicted *K*_eff_ of the model with parallel graphene. This is likely because the modified EMT cannot take into account the size distribution of graphene sheets. In the modified EMT, the length and width were treated as infinite when compared with the thickness of graphene sheets, which failed to take into account the graphene sheet with relatively short length and width (e.g., ~50 nm) [60,61,62]. It should be noted that if given the *K*_eff_ of a specific composite, the interfacial thermal resistance between the nanofillers and the matrix can be estimated by using the developed approach and an inverse calculation procedure. 

## 4. Conclusions

In summary, the developed off-lattice Monte Carlo approach has proved to be accurate as a computational model for heat transfer phenomena and heat transfer mechanisms for multiphase composites with complex structures. The developed approach not only provides a more accurate method to predict the *K*_eff_ of the multiphase composites than existing EMT models, but also offers an effective computational approach to estimate the interfacial thermal resistance between the nanofillers and the matrix. The quantitative findings presented herein showed that multiphase composites with higher *K*_eff_ can be obtained by (a) reducing the interfacial thermal resistances at filler-matrix interfaces; (b) aligning the fillers along the heat flux direction; (c) using fillers with larger interfacial area; and (d) improving the dispersion of fillers to be more uniform rather than forming bundles. Through proper modifications of the geometry and thermal properties of the components in the model, the developed approach may be applied to study the thermal transport properties of other multiphase systems, such as CNT/graphene/polymer composites, graphene stabilized polymer blends, CNT stabilized emulsions and other multiphase organic or inorganic composites.
